# Doing Internet research with hard-to-reach communities: methodological reflections on gaining meaningful access

**DOI:** 10.1177/1468794120904898

**Published:** 2020-02-16

**Authors:** Mareile Kaufmann, Meropi Tzanetakis

**Affiliations:** University of Oslo, Norway; University of Vienna, Austria; University of Oslo, Norway

**Keywords:** Internet research, hard-to-reach communities, credibility, reflexivity, methodology, online research

## Abstract

This article contributes to scholarship on digital sociology by addressing the methodological challenge of gaining access to hard-to-reach online communities. We use assemblage theory to argue how collaborative efforts of human participants, digital technologies, techniques, authorities, cultural codes and the human researcher co-determine aspects of gaining access to online subjects. In particular, we analyse how *credibility* and *reflexivity* are assembled in an online research context. This is exemplified by our own experiences of researching hackers that dispute surveillance and the social embeddedness of darknet drug market users. In this article, we demonstrate the utility of an assemblage perspective for understanding the complexities involved in negotiating access to hard-to-reach communities in digital spaces.

## Introduction

I encrypt all my traffic and almost never write emails that are not encrypted. At the same time, you can find many things about me online, which don’t match my person anymore. I do that so that one cannot find me.

In this quote, the interviewed hacker *=Overview*^1^ describes an approach to avoiding Internet surveillance. The quote, however, also reveals something more implicit: the hacker’s efforts to become invisible to online surveillance also makes it more difficult to reach out to *=Overview* as an interviewee. The ways in which online environments are part of shaping communities that prefer to remain unseen or not attract attention is what this article discusses. We share our experiences and develop a methodological argument about doing Internet research with hard-to-reach communities. Doing research with human subjects is not always as simple as sending out an invitation to participate in a study. Many potential participants cannot be bothered to answer, some may not fit the profile, others would like to keep a low profile because they are marginalized or act on the verge of legality (Hills et al., 2002). It does happen that subjects remain inaccessible to researchers, which can become a methodological challenge (Kristensen and Ravn, 2015). Here, literature tends to focus on sampling the hard-to-reach, as for example with snowball-techniques and chain referrals (e.g. Faugier and Sargeant, 1997). We argue, more profoundly, that the Internet as an increasingly important research environment alters hard-to-reach communities and the issue of gaining access to them. Our ambition is not only to bring the discussion about access into online contexts but also to move away from the narrow focus on sampling. Thus, the article’s central questions are: *How to gain access to hard-to-reach communities online? And how does the specific research environment of the Internet influence sociological methodologies within this field?*

In order to answer these questions, the article draws on a broad understanding of assemblage theory. The idea of the assemblage goes back to Deleuze and Parnet (1977) who describe assemblages as a unity, a symbiosis of many different parts that are connected to each other. The assemblage underlines that dealing with methodological challenges online is neither simply about using the ‘right’ digital tools, nor about bringing methods that have proven useful in offline settings ‘online’. Instead, assemblages frame phenomena and research environments as multiple. They consist of disparate elements: objects, bodies, institutions, actions, ideas, words, etc. These elements may not even work flawlessly together and the way in which they are assembled may change over time, but all the elements still hold together (Buchanan, 2015; Anderson and McFarlane, 2011). In a similar vein, Hodder (2012) uses the perspective of entanglement, which describes relationships between humans and material things. Humans become entangled with objects – in our case global information and communication technologies. While ‘(h)umans make things and the things made by humans make humans’ (Hodder and Lucas, 2017: 136), the relationship between humans and things is not symmetrical in the sense that everything and everyone is ‘irreducibly different’ and the care that humans develop for things is not necessarily reciprocated (ibid.). By understanding phenomena through assemblages and entanglements, we get to see that Internet research is an arena where human and non-human or technological research environments, objects, ethics committees, gatekeepers, tools, connectivity infrastructures, approaches and ideas meet, mingle and influence each other. Assemblage theory reminds researchers to pay attention to the specificity of digital environments and to explore what different elements these spaces are made up of. Such a comprehensive lens also helps researchers to reflect that being hard-to-reach online is different from being hard-to-reach offline. More so, it shows that the *credibility* researchers need to establish in order to gain access to the hard-to-reach, as well as the *reflexivity* that influences the whole research process is equally shaped by the specificity of online environments. As we will show in the next part of the article, many methodological discussions do not sufficiently address these linkages.

We will use our own experiences of researching hard-to-reach communities to illustrate the argument. Kaufmann studies, for example, digital surveillance and how it is challenged by different actors. For one project she conducted qualitative interviews with participants who self-define as hackers. They were invited to join the study as interviewees who have both, a high sense of identification with the Internet, but also the knowledge and interest to challenge the surveillance mechanisms inherent in online technologies. The 22 interviewees were approached via the mailing list of the German Chaos Computer Club with local and international branches. Some interviewees were easily accessible and highly visible subjects who would share their real names and would see no issues in using traceable technologies for the interviews. Other interviewees – after testing the views and knowledge of the researcher – agreed to be interviewed, but ensured their anonymization by using end-to-end encrypted chat software; they would neither reveal their real name, nor voice. Accordingly, the interviewees’ techniques for hacking online surveillance varied largely.

Tzanetakis studies the socio-technical practices with which darknet^2^ drug market users promote coordination on anonymous marketplaces when they face several uncertainties. They cannot be sure about the value of the illicit drugs being traded, nor their profit opportunities. They also don’t know if voluntary agreements are fulfilled in the absence of legal protection and while being prosecuted by the police. This research draws on a multi-sited digital ethnography including 25 encrypted online interviews with cryptomarket users, digital monitoring of drug selling platforms and associated discussion forums, which take into account the self-presentations of vendors as well as the interactions between customers, operators, moderators and vendors. Adults were invited to participate in the study if they had experience with buying and/or selling legal or illegal drugs on cryptomarkets, moderating or operating an anonymous platform. The interviewees were approached via community forum posts and in some cases by the recommendation of gatekeepers. For all encrypted chat interviews, non-traceable software was used, which provides a maximum degree of anonymity and confidentiality.

Our experiences of researching hard-to-reach communities online inform the article’s argument. In the ensuing section, we draw on assemblage theory to describe in what way the Internet is a specific research environment. The characteristics of this environment influence how we access and produce knowledge about online communities. This is followed by a description of those aspects that co-determine hard-to-reach communities online. In the subsequent parts, we use assemblage theory to argue that the methodological concepts of *credibility* and *reflexivity*, too, are co-constituted by the specifics of online environments. Throughout the article, we underline that scientific knowledge is produced collaboratively between researchers, research subjects, assistants, gatekeepers and infrastructures. These findings are summarized and reflected upon in the conclusion.

## The Internet as a specific research environment

Already in 1999, Jones described the Internet as an ‘engine of social change’ (1999: 2), but also as a medium that constantly changes itself (Jones, 1999: 7). Later contributions to Internet studies address more specifically how digital networks are part of altering subjects, practices and concepts. Amongst other things, this includes works about *idioms of practice* (Gershon, 2010), that is the ways in which online communities agree on social uses of technology, but also works about the concept of politics in digital environments (e.g. Kaufmann and Jeandesboz, 2016), the online subjectivity of teenagers (e.g. boyd, 2014), the political and social meaning of hacking (e.g. Coleman and Golub, 2008) or the practice of establishing trust and reputation in encrypted settings (e.g. Tzanetakis et al., 2016; Martin et al., 2019). In line with these works, we foreground how the Internet is part of shaping hard-to-reach communities and the way they act online, but also how the Internet shapes knowledge production per se. For example, it changes how we identify research participants, how we gain access to them and establish credibility. Further, the Internet mediates conversations and observations, which influences the way in which researchers do reflexivity in and about online environments.

The specificity of doing Internet research is nothing new (see Jones, 1999). Most scholars, however, tend to suggest solutions for methodical problems such as sampling, interview techniques, online observation or problems of confidentiality and consent deception (e.g. Stewart and Williams, 2005; Beddows, 2003) – without drawing on a larger framework that grasps the Internet as a research environment. Some scholars begin answering the question of hard-to-involve users, for example, by changing online survey’s quality criteria in order to reach so-called lurkers (Andrews et al., 2010) or by using sampling techniques that identify those who are geographically hard-to-reach (Baltar and Brunet, 2012). As a related field, the issue of Internet research ethics grows, ranging from the safety of the research subjects and the researchers, data protection and ethical-legal challenges to issues of working with publicly available information and informed consent, anonymity and authenticity of subjects (e.g. Ess, 2009; Barratt and Maddox, 2016). Most of these contributions focus – again – on practical or specific ethical challenges in Internet research and do not reflect on the Internet’s impact on the formation of knowledge at large.

Some Internet researchers even take official distance from discussing ‘online interaction more generally’ (Lee et al., 2008: 5). Instead, they

take largely as a given the standards, protocols, mechanisms, and material artifacts that underpin the Internet and allow it to function as a set of ubiquitous, reliable, relatively standardized, and widely shared tools and resources (Lee et al., 2008).

In line with that, some also question the methodological ‘newness’ of Internet studies. Hine (2008), for example, sees no discontinuities between Internet and conventional ethnography and rather frames it as an ‘innovative practice in a recognizable tradition’ (Lee et al., 2008: 13). Such methodological accounts actively refrain from using a theoretical framework to grasp the Internet’s specificities and how these could influence methodological choices.

While we acknowledge the continuities of existing sociological methods, we still think it is crucial to reflect on the specificities of online environments in order to address methodological challenges. Seeing the Internet through the lens of an assemblage speaks to the branch of literature that understands Internet research through the more holistic concept of digital ethnography. Internet research, then, includes both online and offline cultures (Sade-Beck, 2004; Pink et al., 2016). In addition, perceptions of online communities and researchers are central starting points for reflexivity (e.g. Gatson and Zweerink, 2004). Digital ethnography explores the multi-situatedness of communication technology within society, as well as the many forms of embodied online experiences (e.g. Beneito-Montagut, 2011). In line with these approaches, we emphasize that knowledge is always produced in collaboration with all the elements that surround the studied phenomenon. From the perspective of assemblage theory, the Internet is then not just a platform that enables knowledge production between human researchers and human subjects, but the Internet itself is part of knowledge production. The many material aspects and the actions Internet technologies enable play an active role in the creation of knowledge (cf. Kaufmann, 2018a). The Internet is different from analogue tools of information management because it is a set of infrastructures that has traceability and surveillance deeply engrained in its architecture. At the same time, the Internet enables forms of information sharing that redefine time and space. As digital technologies and practices become entangled with ‘human and non-human, organic and inorganic’ (Anderson and McFarlane, 2011: online), online and offline elements of the Internet, they also become part of the reason why some research participants are difficult to access. The affordances of digital environments play, for example, an active role in the cultural codes and practices of the hacker community. Online technologies bring about new modes of disappearing via the ‘creative manipulation of technology’ (*Re-ID*) or by ‘exploring source code in order to trick it. (. . .) One can write one’s own functions to hamper surveillance’ (*LOLveillance*). Further, digital environments not just shape the research subjects and the codices of community, secrecy and credibility they have established, but equally the legal authorities they try to avoid or dispute, and the practices both sides employ. All of these parts co-constitute the phenomenon of hard-to-reach communities on the Internet, and with that, they also contribute to the production of knowledge about them.

The methodological commitment of assemblage theory is here to ‘open’ researchers ‘up’, to bring differences, gaps and dynamics into their focus and to follow ‘an ethos that attends to the social in formation’ (Anderson and McFarlane, 2011: online). Researchers, then, trace the collaborations between the parts of assemblages in order to remain open to the unexpected (Voelkner, 2013: 206). Voelkner also foregrounds that reflexivity is a necessary aspect of assemblage theory as researchers and their instruments equally affect the studied phenomenon (Voelkner, 2013) – an aspect that is crucial in this article. While the study of digital technologies is popular within assemblage or network theories (e.g. Latour, 2005; Bennett, 2010), there are few that have applied assemblage theory to the Internet as such. Hoffman and Novak (2017) have explored the Internet of Things as an assemblage, but have not used the occasion to include methodological considerations about the researcher’s position. Haggerty and Ericson (2000) focus on the influence that the assemblage has on the nature of the studied object, that is surveillance, but they do not embrace the researcher’s role in studying the phenomenon – these are gaps that the following parts address, too.

## Conceptualizing hard-to-reach communities online

Communities that are difficult for researchers to access are traditionally understood as peripheral communities, sometimes even with a clear reference to social fringes and deviance (Lambert and Wiebel, 1990). In this article, the deviance of hard-to-reach communities is not their central determinant. The communities we explore here may very well not be average Internet users and could thus be understood as peripheral, marginalized or stigmatized. Yet, what makes them hard to reach is an assemblage of many elements.

### Hard-to-reach communities online are multi-sited communities

They are, for example, not geographically in one location or on one site, but their members interact in various places (Pink et al., 2016), online and offline. In order to study darknet drug market communities, for example, we need to look at multiple platforms on the darknet, discussion forums, vendor shops and sites providing information on the ecosystem, all of which rapidly change over time (Tzanetakis, 2018). By using ‘communities’ as a unit of analysis we consider the social glue of members’ relationships across sites, but also the political implications of collective terms since the term community is not as clear-cut as, for example, the term ‘population’ would suggest (Pink et al., 2016).

### Most members of the online communities we study have technological proficiency

A crucial determinant of the communities we study is how its members navigate the online environment they act in. They have knowledge, skills, awareness or insights into technological functions that the digital mainstream does not have. For example, hacker *Re-ID* finds it important to

learn about possibilities and dangers of surveillance technologies. I hack because I need a larger picture of the technology I’m dealing with. (. . .) I have a certain need for protection. I want to safeguard my privacy online.

Technological skills also characterize the darknet drug market community. Here, we see even more that the ability to act on multiple sites is central to members of that community. The cryptomarket community uses digital technologies to distribute illicit drugs in a systematic way, with which they break the law in most jurisdictions. Darknet drug market user *Velopin* puts in a nutshell:

I use anonymizing technologies to get exactly the products for money I want to put in my body, they are of better quality than most stuff on the streets and without interference of a state. I have complete sovereignty of my body. It’s nobody else’s business what I do with my body.

Being hard to reach online is central to both communities, but for different reasons. While some hackers want to safeguard privacy and question technological standards, it seems that some members of the darknet drug market community question dominant drug control models.

### Hard-to-reach communities online are also shaped by technologies and techniques

Whether members of hard-to-reach online communities are careful, shy, distrusting or eccentric users of digital networks, they actively seek to become untraceable or simply have the ability to disappear from online view. As such, hard-to-reach communities online are co-determined by the Internet’s infrastructure and the digital techniques of disappearance and non-traceability they put to use. Practically, they may communicate in peer-to-peer networks, use encryption, obfuscation (Brunton and Nissenbaum, 2011; Martin et al., 2019) and other strategies of countering or bypassing surveillance or law enforcement (Kubitschko, 2015). Hacker *AceOfPlays*, for example, summarizes:

I encrypt my emails, I leave my cell phone at home or don’t use the phone. I may go offline to not leave any data traces and I use encrypted apps only. If I really want to be left alone online, I use TOR, but even there it is never clear who operates the entry and exit nodes.

Encryption software allows its users to conceal their identities and locations and therefore to hide from and become hard to reach for the outsiders. This is exemplified by the use of encryption technologies like *The Onion Router* (Kaufmann, 2018b; Tzanetakis et al., 2016). These techniques are associated with their own benefits and disadvantages – dependent on their purpose, which also adds an ideological level to the choice of technique (Kaufmann, 2018b).

### Social labels associated with specific types of online behaviour co-constitute the hard-to-reach

What stereotypically comes to mind when studying the hard-to-reach online are research subjects with illicit intentions, but also privacy advocates, activists, political refugees or simply those who enjoy playing with surveillance systems (Kaufmann, 2018b). Despite the fact that the reasons for disappearing online are this manifold, disappearance tends to be associated with hiding (Tzanetakis, 2018) or with weak forms of protest as its users want to enjoy online services without contributing to their functioning (Brunton and Nissenbaum, 2011: online). Whether they truly apply or not, such pejorative labels are also part of the assemblage and further complicate access to these groups.

### The specificities of online communities determine whether we gain access or meaningful access

An experience from Tzanetakis is that communities who are involved in darknet drug markets include customers, dealing customers, small-level dealers, high-level dealers, moderators, administrators of platforms and information hubs – some of them are easier to talk to than others. Relying exclusively on the most accessible members of online communities also determines the research results. The community member *Hyponaup* says: ‘The smart ones won’t talk, and the ones who do talk are either foolish and not long for this world or are doing it as PR/advertising and you can’t trust what they say, don’t you?’. Hence, recruiting a member of a hard-to-reach community into a research project does not necessarily entail meaningful access. Some communities regulate access in surprising ways. While trying to reach hackers that question online surveillance, Kaufmann experienced that the addressed did not answer *her* questions, but instead she needed to answer *theirs*. As a response to her call for interviewees, she was tested to figure out what her ideological standpoints would be. One person who answered the call for participation asked: ‘What is the relationship between corporate surveillance and the state?’ Kaufmann needed to give the interviewee an acceptable answer before being allowed to gain further access. For others, she needed to install specific types of software in order to interview them and configure the software while they communicated. When she reflected on these experiences together with *DataD14709*, the interviewee said:

Well, you know, within our community there is a thing called the social captcha test. In the same way that you’d complete a captcha test online, you also have to pass a captcha test as a human being, a hacker, a member of a social network. Every community has their own test and you have to find out how to do it. (. . .) In hacker communities you will mostly experience technical captchas. We want to find out how much IT knowledge this person has, whether we like this person. Does the person do activities that we do, is the person interested in things we are interested in?

Passing a social entry barrier to gain meaningful access to research subjects is part of most qualitative research projects. The social captcha test may be the equivalent for research in online environments. While the original captcha test was developed to tell humans apart from robots that try to access online information, the social captcha test is a moniker for procedures and codices that online subjects put to use to figure out if they want to allow for further communication. For us, the identity-statement of social captcha tests is a starting point for reflections on gaining access to online communities, but also about doing Internet research at large.

## Assembling credibility to gain meaningful access

In our experience, one of the first steps of gaining access to hard-to-reach communities can be described as the carefully composed moment of credibility. The concept of credibility has been discussed in methodological literature in terms of sampling techniques (Cohen and Arieli, 2011) and the trustworthiness of the study (Denzin and Lincoln, 2005). Inspired by Aradau and Huysmans (2018), we want to examine what it means to assemble credibility vis-a-vis research subjects in an online environment. Indeed, credibility is not determined by the researcher alone, but it is a negotiation and a multi-faceted phenomenon. This position emphasizes that ‘all propositions have to win credibility, and credibility is the outcome of contingent social and cultural practice’ (Shapin, 1995: 257). Credibility, then, is assembled – sometimes intentionally, sometimes unintentionally – but always within a specific context.

### Credibility requires commitment

Credibility can be conceptualized as an investment of time and resources to build a trustworthy relationship between the researcher and hard-to-reach subjects. Mayorga-Gallo and Hordge-Freeman (2017) distinguish between cultural and professional credibility. While the professional credibility refers, for example, to the researcher’s institutional affiliation and the use of methods, cultural credibility is linked to signalling openness and familiarities with the specific community. However, we emphasize that each type of credibility does not work within every context. For gaining access to the community of darknet drug market users, Tzanetakis set up a study website and described the aim of the research project in plain language. Moreover, she highlighted her university ties by placing the website within the university servers and using the university’s logo. In addition, she listed scholarly publications, public presentation and media appearance on the study website in order to signal the community that she has experience in the field. Providing this information assisted cryptomarket users in assessing her cultural knowledge and making sure that their time spent on interviews was well-invested. *Haugemod* confirmed:

Yes, your name appeared on Deepdotweb [a former darknet market information website] and I checked your website and read some media reports about you. I don’t want to waste my time on newbies [newcomers].

Furthermore, Tzanetakis published her institutional e-mail-addresses, public encryption keys and named the software for conducting online interviews on both forums and the study website. Making these technical codes available online turned out to be valuable in order to signal the hard-to-reach communities that she speaks their language and is sensitive to their risk of law enforcement activities. Thus, her credibility was geared towards a specific community. It was partly the result of aspects she could influence, but also the product of perceptions of culture, the technologies in use and the cultural codes of the community.

### Credibility also involves the researcher’s position as an insider/outsider

The status of being an insider/outsider to a specific online community influences whether one gets meaningful access to an online community. As hacker *DataD14709* explains from experience: ‘If you want to enter a community you have to check them out first. You really can’t botch that captcha test up.’ The status of an in- or outsider involves many aspects. It depends on the digital technologies put to use to reach out to subjects, as well as the legal, social and cultural understanding of the research subjects’ activities. Becoming a cultural insider means that the researcher shares characteristics, roles or experiences with the studied community (Dwyer and Buckle, 2009). Most likely, the researcher is both, an insider and outsider at the same time.

Following Adler and Adler (1987), Tzanetakis adopted a peripheral membership role by maintaining a transparent position as a researcher and by acquiring knowledge about the community’s culture, such as the technologies in use, the functioning of anonymous platforms, the subjects’ vulnerability vis-à-vis law enforcement and fraud, the specific language and vocabulary of abbreviations. In addition, she established contact to gatekeepers (e.g. moderators) who were able to signal the online community that her research is reasonable before posting requests for participation. This approach allowed her to position herself between the binaries of insider or outsider: in some cases, she was perceived as a cultural outsider (no direct involvement) while in others as a cultural insider (technological knowledge). Different from peripheral membership, an active membership would include, for example, volunteering as an administrator of a drug harm-reduction forum (Barratt and Maddox, 2016). Complete membership means that researchers are fully immersed in the social world studied. Becoming a complete member within the darknet drug market community would raise concerns about research ethics and can in some cases even put the scientific basis of the research results into question, but it enables the most advanced access to the studied community. Here, the need to balance the many facets of establishing credibility becomes obvious.

Researchers can also take a covert role vis-à-vis the community they seek to study. Here, the research subjects are not informed about the research and the utilization of any documentation (Paechter, 2013). While in some cases, a disclosure of the researcher’s role might restrict the access to the community, controversial ethical implications of covert online research, like the protection of privacy, anonymity, accountability and consent have been discussed (e.g. Ess, 2009). The covert role may also pose ethical challenges to the aspect of credibility via-à-vis oneself. Closely related to the covert role is the ‘lurker’. In contrast to face-to-face qualitative research where everyone present is visible, online research enables researchers to observe interactions without overtly participating in it (Paechter, 2013). A lurker may, for example, sign into services, read messages and posts, but does not post messages. With the lurker in online environments, the nature of participant observation changes as it allows to observe without informing the observed and without their consent and also without having clarity about the insider/outsider status of the researcher. It works on the assumption that lurking does not alter the behaviour of the online subject (Ferguson, 2017). However, most qualitative researchers are active parts of the field they are studying and, accordingly, the researcher’s context (gender, class, ethnicity, sexuality and people on the sociocultural margins) equally shapes the accessibility of and knowledge produced about their research subjects (Angrosino, 2005). Establishing the status of a researcher is always done as part of the larger assemblages in which research subjects are situated and most likely, researchers combine different roles.

### Credibility is about the safety of the research participants

When members of hard-to-reach communities participate in research projects they can face social, legal, psychological or physical risks. This was the case with Tzanetakis’s research on darknet drug market users that included sensitive research questions about behaviour for which the users could face legal consequences. Such questions can make members of the community more hesitant to identify themselves to her or any researcher. The safety of research participants is even more relevant in online environments as communication usually leaves traces (e.g., IP addresses, cookies, account registrations and geolocation, cf. Kaufmann and Jeandesboz, 2016) through which the users can be identified. According to hacker *Numbercruncha*, ‘there is no unsurveilled space online. There may be space where you can be anonymous. But even in anonymous networks officials and secret services try to identify you.’ For this reason, both of the communities we discuss here are keen to use encryption software and specific settings to conduct interviews safely. Taking precautionary measures to safeguard the rights and well-being of the research participants, regardless of their stigmatization, also plays into credibility (cf. Markham and Buchanan, 2012). For conducting interviews with darknet drug market users, Tzanetakis used non-traceable software and took care not to include or collect identifying information (e.g., names, locations) when storing the interview protocols. Credibility, then, is assembled as a performance by the researcher, which includes the use of up-to-date technologies in order to signal the research subjects that risks related to participation are adequately addressed. However, since digital technologies are constantly evolving, new flaws in programs are identified. The Internet is a dynamic research environment that necessitates ongoing positioning of its researchers vis-a-vis their subjects.

### Credibility also implies the researcher’s safety

Often, researchers who opt for transparency about their identities and activities are themselves at risk. In online environments, the visibility of the researcher differs from face-to-face research settings as Internet research can attract trolling behaviour online, but also offline harassment and threats of violence (e.g. Buckels et al., 2014). Reactions on Tzanetakis’s own posts for online interview participation within the darknet market community included appreciation by some people, but also sceptical comments by others. However, none of them included implicit or explicit threats. On the practical level, Tzanetakis followed preparatory measures suggested by Barratt and Maddox (2016). These included securing passwords for all digital accounts, emotional preparation for trolls, engagement with gatekeepers of the community (e.g., forum moderators, valued community members) and storage of interview protocols on encrypted devices before entering the field. Here, credibility is assembled via the researcher’s awareness about possible unintended consequences of doing research overtly with hard-to-reach online communities.

## The dynamic relations that influence reflexivity in the research process

The work of assembling credibility foregrounds the many relations that are at work in the process of gaining access. And yet, there are many more interrelations at play. For example, the techniques used to connect to each other and the related digital research tools are equally active parts in the negotiation of access. A methodological concept that assesses these relationships is reflexivity. It describes the process of becoming aware of dynamics of influence. As such, reflexivity can be a part of any step in the research process.

### Reflexivity is thinking about situatedness

Often, reflexivity is tied to the moment of self-referral in order to situate the author(s) within the research project. Feminist readings of reflexivity suggest using it to locate power relations in fieldwork (England, 1994). This became relevant, for example, when both of us who identify as cisgender women, got entangled with a predominantly male community of hackers or darknet drug market users. In a broader sense, reflexivity also means to interrogate the methods one uses, to think about research questions in larger contexts, as well as to reflect upon alternative paths to do research (Markham and Baym, 2009: 142). Reflexivity is thinking about the social, historical, institutional, ideological, cultural and methodological situatedness of a research project, a researcher, a research subject, object or tool (Guillaume, 2015). Researchers tend to take these positions for granted and keep them at an ‘unthought stage’ (Bourdieu, 1994: 217). Tracing the assemblages of a research project is then a moment of excavation. It is about pointing to those relations and conditions that usually get overlooked and it brings researchers to that which they not get to produce (Adams and Holman Jones, 2011). Yet, tracing these assemblages also makes researchers walk new avenues. It decentres the researchers; it leads away from ‘ourselves’ towards those who receive of ‘us’, who give us access and allow us to speak to them. Reflexivity introduces researchers to the many objects and circumstances that enabled the production of knowledge.

### Reflexivity, then, is to acknowledge that knowledge is produced collaboratively

That reflexivity includes any party in the study – researchers, participants, assistants, gatekeepers and all of them with their diverse institutional backgrounds – is a well-established insight and has a strong tradition in feminist research (cf. Caretta, 2014). Reflexivity is to consider how research is

‘happening between,’ within the negotiated relations of whose story is being told, why, to whom, with what interpretation, and whose story is being shadowed, why, for whom, and with what consequences (Fine, 1998: 35).

The knowledge produced in the projects that we, the authors, conducted is co-constituted by the various ideological standpoints about the researched matter, which influence how credibility and access were established. Our research is shaped by requirements and ideas of funding bodies that set the framework for conducting research, the ethical guidelines that the project needs to comply with, the software configurations that allow for specific forms of online communication with the researched subjects, the screen names that online subjects use and the specific culture of anonymity this creates. Thus, reflecting on a project’s assemblage also leads researchers to become sensitive to those influences that not necessarily lie in their hands. There are many levels of observing and being observed (i.e. by research subjects, by ethics authorities and by representatives of specific ideologies) and there are many levels of enabling and being enabled when we produce knowledge (Guillaume, 2015: 193). Reflexivity, then, does not just include reflections on the ways in which researchers gain access to the subject, but it also leads researchers to the research subject who opened up and gave answers.

### Reflexivity means finding ways of letting the research subjects surprise you

Reflexivity is a key instrument to allow research subjects speak up, to let them overthrow our assumptions about them. This process includes reflections on the preconceptions that shape, for example, interview guides. Further, it requires a dynamic dialogue with research subjects and to let them engage with us, to let them surprise us and broaden the repertoire through which we see the world. For example, after a long talk about the methods of questioning surveillance that hackers also use to express their identity, some interviewees surprised Kaufmann. In response to the question ‘As a conscious Internet user, is it important to act differently from the mainstream, the masses?’ *Panoptipwned*, said:

For me, it’s not important to be different from the mainstream. Actually, I hope that hackers’ knowledge about surveillance and our techniques to deal with online veillance will become the mainstream.

These dialogues were also shaped by the specifics of the research environment Internet. Since Kaufmann used online programs for the interviews, the potential interviewees were aware of the fact that everything they said online could also be traceable. This caused a certain scepticism to talk to her. Her’s solution was to negotiate with every interviewee the interview-tool that would allow for the most meaningful access. Each solution was determined by what the interviewee considered the most and least intrusive software for conducting the interviews online. The reasoning about which tool would be the most trustworthy implied theories, insights and ideologies of what the Internet was, how online surveillance would be implemented and by whom. On Kaufmann’s side, this required a flexibility in approaches for gaining access. It involved learning about new technologies, communication channels, as well as specific arguments and views on what counts as trustworthy online technology.

It becomes clear that all of these aspects were influenced by the Internet. Reflecting on the functionality of technological objects, as well as the related political and ideological standpoints of the interviewees was here indispensable. Reflexivity, thus, may lead researchers to interviewees that ‘speak up’, but it also helps researchers to work with these surprises. However, research subjects are not the only ones who speak up in collaborative processes.

### Reflexivity also needs to apply to research tools

Methodological instruments or research tools can also speak up, surprise us or guide our research without us noticing. They are the result of decisions, opinions and disciplinary routines. They arise from interactions between human users and non-human technologies and techniques. Because of that, research instruments and the knowledge they produce are inherently social and normative (Aradau et al., 2015). Accordingly, reflexivity is the attempt to become aware of the way in which research instruments shape the research process. This could include anything from research design to concrete research tools, as for instance the software programs used to conduct interviews online. In her project on hacking online surveillance, Kaufmann carefully negotiated the use of specific tools to conduct the interviews in advance (as described above), but these tools also acted back in the sense that some of them were unintuitive to install and had technical glitches, which at times interrupted the communication. These glitches complicated access and shaped the flow of the interviews. For her ethnographic research on darknet drug markets, Tzanetakis used anonymizing software to access, for example, specific marketplaces. However, establishing a connection with a darknet network usually takes more time than other browsers, which is a side effect of disguising the location of its users. This is how research tools are part of shaping the research process and its outcomes at a practical and technical level.

Yet, the same tools also carry political meaning, precisely because they were chosen by the interviewees to minimize data traceability and maximize anonymity. The limitation of traceability is relatively well-executed by open-source and niche-oriented software with non-standard communication protocols. This is different from popular commercial software that supports instant messaging and videoconferencing. In fact, some hackers or darknet market users would not have participated in the study or would have given different answers if they were interviewed via products that trace and store data. These examples illustrate how software can influence the knowledge produced within a research project. In addition, the open source software used to conduct interviews are normatively loaded. Especially, open source encryption software, for example, are publicly portrayed as a tool that can be used for illicit activities. The social labels attached to such tools shape the type of access one can get to online communities when using these tools. They determine which communities will answer a call and they influence the answers of the interview subjects. Reflexivity, then, is the examination of how online research tools may speak up and how they shape access to hard-to-reach communities and knowledge production in dialogue with them.

## Conclusions

Passing a social entry barrier to gain meaningful access to research participants is something that most researchers experience. Online, the social captcha test illustrates that gaining access not only lies in the researcher’s hands, at the same time being hard-to-reach not only refers to the research subjects alone. The environments in which subjects and researchers move, as well as the tools researchers use to gain access to, observe and interview subjects are part of a bigger assemblage.

The social captcha test signals here the dialogical dynamics involved in establishing credibility to gain meaningful access. Credibility is not just something that a researcher obtains, but the technologies, techniques, institutions and cultural codes of the community under study are part of it. To build a trustworthy relationship between the researcher and the participants, for example, the researcher needs to understand the cultural codes of the online community. It also includes the appropriate use of communication technology to safeguard the well-being of research participants. Further, a social captcha test is a reminder that Internet research has to do with the ‘I’ – with situatedness and reflexivity. The ‘I’, however, should not mislead researchers to limit their reflections to self-reflexivity. The ‘I’ gets entangled with diverse technical components when doing Internet research. In this article, we emphasized the many different elements that are part of knowledge production online. In doing so, we developed the argument that the ‘I’ is also a reflexive ‘We’, an assemblage of the many human and non-human elements that surround the phenomena we study. Reflexivity is an invitation to trace these assemblages, to be surprised, to let these elements speak up, to be directed towards the unexpected, but also the silences and ultimately that which cannot be known (cf. Adams and Holman Jones, 2011).

In reference to our own empirical work, we illustrated that such an assemblage includes the technologies and practices that allow subjects not to be seen online, the societal labels that are attached to such practices and communities, the screen names and the culture of anonymity they produce, the researcher’s status of credibility, the related range of ethical, social or technical procedures that establish a researcher as a community’s in- and/or outsider, the specific software that needs to be installed to get in touch with subjects, the negotiations about the choice of software, as well as their glitches. All of these aspects are part of constituting both, the hard-to-reach and how access to them can be gained. Delimiting such a research assemblage is difficult, but also not necessary. Rather, we argue that considering the assemblage of hard-to-reach online communities brings the entanglements to the fore, which are constitutive of the knowledge we produce about the phenomena we seek to understand.

## Notes

1. For all quotes by interviewees presented in this article, pseudonyms are used. Any similarities with existing handles are unintentional.

2. The darknet is a hidden part of the Internet accessible only with anonymizing software.
